# Urine Dipstick Proteinuria and Cholelithiasis

**DOI:** 10.2188/jea.JE20210281

**Published:** 2021-11-05

**Authors:** Shih-Wei Lai

**Affiliations:** 1 Department of Public Health, College of Public Health, and School of Medicine, College of Medicine, China Medical University, Taichung, Taiwan; 2 Department of Family Medicine, China Medical University Hospital, Taichung, Taiwan

Dear editors,

A retrospective cohort study conducted by Park et al published in the *Journal of Epidemiology* reported that dipstick proteinuria (≥2+) was associated with an increased hazard of cholelithiasis in Korea (adjusted hazard ratio 1.46; 95% confidence interval, 1.09–1.96).^1^ Some points are discussed. First, urinary protein changes dynamically over time. The majority of proteinuria detected using urine dipstick are benign and no related morbidity can be found, such as dehydration or orthostatic proteinuria.^2^ Previous studies suggest that urine dipstick is a not accurate tool for screening proteinuria.^3^^,^^4^ In Park et al’s study, the measurement of proteinuria was detected using urine dipstick based on only one single voided urine sample. No confirmation test was performed. The accuracy of proteinuria is doubtful. Therefore, the interpretation of the association between dipstick proteinuria and cholelithiasis risk found in Park et al’s study should be cautious. Second, the eGFR data were available in Park et al’s study. Thus, the stage of chronic kidney disease can be defined. If dipstick proteinuria is associated with chronic kidney disease, the hypothesis raised by the authors that the presence of renal disease reflected by dipstick proteinuria might be partially supported. The readers will understand the association between proteinuria, chronic kidney disease, and cholelithiasis risk more clearly. Third, the probability that patients with dipstick proteinuria (≥2+) truly had cholelithiasis was only 2.4% during the cohort period in Park et al’s study.^1^ That is, the positive predictive value for cholelithiasis using dipstick proteinuria (≥2+) was low. Dipstick proteinuria is not a cost-effective tool for screening cholelithiasis. Fourth, whether or not asymptomatic people with proteinuria should undergo routine abdominal ultrasound examination for detecting cholelithiasis is still uncertain. However, if an acute abdomen is presented, it is a reasonable arrangement to undergo abdominal ultrasound examination. Finally, I agree with the authors’ conclusion that the long-term association between proteinuria, renal function, and cholelithiasis risk needs further investigation.
